# Optimization of Enzymatic Hydrolysis and *Leuconostoc mesenteroides* MKJW Fermentation of *Gryllus bimaculatus* Using Response Surface Methodology

**DOI:** 10.3390/foods15203684

**Published:** 2026-10-16

**Authors:** Minji Cho, Misook Kim

**Affiliations:** Department of Food Science and Nutrition, Dankook University, Cheonan 31116, Republic of Korea; bluepill0103@naver.com

**Keywords:** degree of hydrolysis, Box–Behnken design, α-glucosidase inhibition, microbial viability, edible insect protein

## Abstract

*Gryllus bimaculatus* (GB) is a promising sustainable protein source, and its bioprocessing performance is strongly influenced by processing conditions. Enzymatic hydrolysis and lactic acid bacterial fermentation represent complementary approaches for modifying GB components and developing value-added products. In this study, response surface methodology (RSM) with a Box–Behnken design was used to optimize two GB bioprocesses: Neutrase-mediated enzymatic hydrolysis and fermentation with *Leuconostoc mesenteroides* MKJW. For enzymatic hydrolysis, GB concentration, Neutrase concentration, and hydrolysis time were evaluated using degree of hydrolysis (DH) as the response. The model-derived condition associated with a high predicted DH within the tested range was 20% (*w*/*v*) GB, 136.2 U/mL Neutrase, and 31 h, under which the experimental DH was 78.59% compared with a predicted value of 87.03%. For fermentation, GB concentration, sucrose concentration, and fermentation time were simultaneously evaluated using viable cell count and in vitro α-glucosidase inhibition (AGI) as responses. Multi-response optimization identified 7.1% (*w*/*v*) GB, 6.7% (*w*/*v*) sucrose, and 27 h as the model-derived condition associated with simultaneously high viable cell count and AGI within the tested range, yielding experimental values of 9.94 log CFU/mL and 92.94% AGI compared with predicted values of 9.98 log CFU/mL and 86.57%, respectively. Overall, RSM enabled the quantitative identification of distinct process drivers for GB hydrolysis and fermentation and the experimental evaluation of model-derived operating conditions, providing a process-level basis for subsequent compositional, mechanistic, and functional characterization.

## 1. Introduction

Global demand for animal protein continues to increase while land availability, environmental impacts, animal-welfare concerns, and resource constraints intensify the search for alternative protein sources. Edible insects have therefore attracted attention as potentially sustainable food and feed ingredients [1]. They generally contain high levels of protein together with minerals, vitamins, and nutritionally relevant fatty acids, and many edible-insect proteins provide essential amino acids in proportions compatible with human nutritional requirements [2]. In addition, insect production can offer advantages over conventional livestock systems in feed conversion and resource use [3].

GB is an edible cricket species with established use as a food and feed ingredient, and its nutritional value has been documented in previous studies [4,5]. GB contains substantial amounts of protein with a balanced amino acid profile and also provides unsaturated fatty acids and minerals [6]. These characteristics make GB a suitable substrate for bioprocessing strategies aimed at modifying its protein components and developing value-added ingredients.

Proteolytic hydrolysis cleaves peptide bonds and generates peptides and free amino acids of different molecular sizes. Enzymatic hydrolysis is therefore widely used to modify food proteins and can generate peptide fractions with biological activities, depending on the substrate, enzyme, and processing conditions [7,8]. Degree of hydrolysis (DH) is a process indicator that reflects the extent of protein hydrolysis, but DH alone does not establish the composition or bioactivity of the resulting hydrolysate. Previous studies have reported antioxidant and anti-inflammatory activities in hydrolysates from edible-insect proteins [9]. Hydrolysis outcomes are nevertheless strongly affected by enzyme specificity, substrate concentration, reaction time, temperature, and related process variables. Optimizing these variables is therefore important for identifying processing conditions associated with the desired extent of protein hydrolysis.

Lactic acid fermentation is a traditional food-processing and preservation strategy in which microorganisms convert fermentable carbohydrates into lactic acid and other metabolites. Depending on the substrate and microbial strain, fermentation can influence product stability, sensory properties, and biochemical composition [10]. Microbial proteases and peptidases may also contribute to the breakdown of food proteins into lower-molecular-weight peptides and free amino acids during fermentation [11]. Moreover, fermentation performance depends on multiple interacting factors, including substrate composition, available carbohydrate, microbial strain, and fermentation time, making simultaneous optimization of these variables important for process development.

Response surface methodology (RSM) is a multivariate statistical approach commonly used in food process optimization to model the effects of multiple independent variables on one or more responses. It can be used to identify interactions, estimate quadratic effects, and determine processing conditions associated with desired responses [12]. Among commonly used response-surface designs, the Box–Behnken design (BBD) efficiently estimates second-order models while avoiding experimental combinations in which all factors are simultaneously set at their extreme levels [13]. This approach is particularly suitable for bioprocess optimization because multiple processing variables can be evaluated simultaneously while reducing the number of experimental runs required relative to full factorial designs.

Previous studies have demonstrated that fermentation or enzymatic processing can modify the nutritional and functional properties of edible insects, including GB [8,9,14,15]. However, the effects of interacting processing variables on Neutrase-mediated GB hydrolysis and the relationship between microbial growth and AGI during GB fermentation have not been fully characterized. The present study therefore quantitatively evaluated interacting process variables and experimentally evaluated model-derived conditions for two distinct GB bioprocesses. For enzymatic hydrolysis, GB concentration, Neutrase concentration, and hydrolysis time were evaluated using DH as the response. For fermentation with *Leuconostoc mesenteroides* MKJW, a kimchi-derived strain previously reported to exhibit α-glucosidase-inhibitory activity [16], GB concentration, sucrose concentration, and fermentation time were evaluated using viable cell count and α-glucosidase inhibition as the responses. Viable cell count and AGI were evaluated simultaneously to determine whether conditions favoring microbial growth were also associated with the functional screening response. The model-derived conditions associated with high predicted responses within the tested ranges were subsequently evaluated experimentally.

The present study provides process-specific quantitative evidence regarding the effects of processing variables on GB hydrolysis and fermentation. Notably, the results revealed distinct patterns of association between processing variables and microbial growth or α-glucosidase inhibition during fermentation. These findings provide a process-level basis for subsequent compositional, mechanistic, and functional characterization.

## 2. Materials and Methods

### 2.1. Materials and Preparation of Gryllus bimaculatus Powder

Adult GB were purchased from an edible insect farm (Cricket Farm Co., Hwaseong, Republic of Korea) after hot-air drying at 70 °C for 10 h. The dried GB samples were pulverized using a grinder (SMX-M41KP, SHINIL, Cheonan, Republic of Korea), sieved through an 18-mesh sieve, further dried at 55 °C for 8 h in a drying oven (JSOF-150, JS Research Inc., Gongju, Republic of Korea), and stored at −18 °C until use. Crude protein content was determined by the Kjeldahl method according to AOAC International [17], using an N-to-protein conversion factor of 6.25, and crude ash content was determined by incineration at 550 °C to constant weight according to Quirino et al. [18]. Chitin content was estimated gravimetrically after sequential demineralization with 1 M HCl (Samchun Pure Chemical co., Ltd., Pyeongtaek, Republic of Korea) (1:10, *w*/*v*; 100 °C, 100 rpm, 30 min) and deproteinization with 1 M NaOH (Duksan Pure Chemicals Co., Ltd., Ansan, Republic of Korea) (1:10, *w*/*v*; 80 °C, 100 rpm, 24 h), with modifications based on Liu et al. [19]. The recovered chitin was washed to neutral pH, dried to constant weight, and expressed as a percentage of the initial GB dry weight. The GB powder used in this study contained 70.16 ± 5.08% crude protein, 5.32 ± 0.04% crude ash, and 6.29 ± 0.20% chitin on a dry-weight basis. The lactic acid bacterium used for fermentation was *Leuconostoc mesenteroides* MKJW (KCTC 14459BP). Neutrase (Novozymes®, Bagsværd, Denmark) was used for protein hydrolysis. α-glucosidase from *Saccharomyces cerevisiae* (Sigma-Aldrich, St. Louis, MO, USA) was used for the α-glucosidase inhibition assay.

### 2.2. Experimental Design and Response Surface Methodology

Response surface methodology (RSM) based on a Box–Behnken design (BBD) was used to optimize enzymatic hydrolysis and lactic acid bacterial fermentation of GB. Each three-factor, three-level design comprised 17 experimental runs, including five replicates at the center point. The coded and actual levels of the independent variables for both processes are presented in Table 1. Experimental designs and model fitting were performed using XLSTAT-Student version 2024.4.1.1425 software (Addinsoft, Paris, France) in Microsoft Excel version 16.0.18526 (Microsoft Corporation, Redmond, WA, USA).

### 2.3. Optimization of Enzymatic Hydrolysis of Gryllus bimaculatus

For enzymatic hydrolysis, GB concentration, Neutrase concentration, and hydrolysis time were used as independent variables, with degree of hydrolysis (DH) as the response. GB concentrations above 20% (*w*/*v*) were not included because preliminary handling observations showed that the suspensions became visibly more viscous and could not be mixed homogeneously under the hydrolysis conditions used. A mixture containing GB at the specified concentration (% *w*/*v*) in 0.07 M sodium phosphate buffer (pH 6.0) was autoclaved at 121 °C and 15 psi for 30 min using an autoclave (PVC-60, LAB HOUSE, Pocheon, Republic of Korea) and then cooled. Neutrase was added to obtain the designated enzyme activity (U/mL), expressed as units of enzyme activity per milliliter of the total reaction mixture. Hydrolysis was performed at 55 °C and 100 rpm for the specified reaction time in a shaking incubator (SI-121, LAB HOUSE, Pocheon, Republic of Korea). After hydrolysis, the supernatant and residue were separated by centrifugation at 4355× *g* and 4 °C for 30 min (Avanti J-E, Beckman Coulter, Brea, CA, USA), and the supernatant was filtered through Whatman No. 1 filter paper (Cytiva, Marlborough, MA, USA).

### 2.4. Optimization of Leuconostoc mesenteroides MKJW Fermentation of Gryllus bimaculatus

For fermentation, GB concentration, sucrose concentration, and fermentation time were used as independent variables, with viable cell count and α-glucosidase inhibition (AGI) as the responses. The fermentation medium consisted of the designated concentration of GB in a glucose-free modified MRS-type basal medium [20] containing 0.4% (*w*/*v*) yeast extract (Gibco, Thermo Fisher Scientific, Waltham, MA, USA), 0.5% (*w*/*v*) sodium acetate (Junsei Chemical Co., Ltd., Tokyo, Japan), 0.2% (*w*/*v*) ammonium citrate (Samchun Pure Chemical Co., Ltd., Pyeongtaek, Republic of Korea), 0.2% (*w*/*v*) dipotassium phosphate (Duksan Pure Chemicals Co., Ltd., Ansan, Republic of Korea), 0.02% (*w*/*v*) magnesium sulfate (Junsei Chemical Co., Ltd., Tokyo, Japan), 0.005% (*w*/*v*) manganese(II) sulfate monohydrate (Tokyo Chemical Industry Co., Ltd., Tokyo, Japan), 0.1% (*v*/*v*) polysorbate 80 (Duchefa Biochemie B.V., Haarlem, The Netherlands), 0.8% (*w*/*v*) beef extract (Gibco, Thermo Fisher Scientific, Waltham, MA, USA), and 1% (*w*/*v*) proteose peptone (MB Cell, Seoul, Republic of Korea). The basal medium was based on the MRS-type formulation and was maintained at a fixed composition to provide a consistent growth-supporting background for *L. mesenteroides* MKJW. Glucose was omitted because sucrose concentration was included as an independent variable in RSM design. Accordingly, the remaining basal medium components were kept constant, while GB concentration, sucrose concentration, and fermentation time were varied experimentally. The GB-containing medium was autoclaved at 121 °C and 15 psi for 30 min and then cooled. Sterile sucrose (Duksan Pure Chemicals Co., Ltd., Ansan, Republic of Korea) was subsequently added to the designated concentration. *L. mesenteroides* MKJW was inoculated at an initial cell density of approximately 6 log CFU/mL. Fermentation was carried out at 30 °C and 100 rpm for the specified time. After fermentation, the supernatant was obtained by centrifugation at 4355× *g* for 30 min at 4 °C and filtered through Whatman No. 1 filter paper.

### 2.5. Degree of Hydrolysis

Degree of hydrolysis (DH) was determined using a ninhydrin-based method adapted from Zhang et al. [21] and Sun et al. [22]. A 0.2 mL aliquot of ninhydrin solution, consisting of 2 g ninhydrin (Daejung Chemicals & Metals Co., Ltd., Siheung, Republic of Korea) and 0.3 g hydrindantin dihydrate (Tokyo chemical Industry Co., Ltd., Tokyo, Japan) dissolved in 75 mL dimethyl sulfoxide (Duchefa Biochemie B.V., Haarlem, The Netherlands), and 25 mL of 4 M sodium acetate buffer, was mixed with 0.2 mL of the sample and vortexed using a vortex mixer (Vortex-Genie 2, Scientific Industries, Inc., Bohemia, NY, USA). The mixture was then heated in boiling water for 15 min. After cooling, 1 mL of 50% (*w*/*v*) ethanol (Samchun Pure Chemical Co., Ltd., Pyeongtaek, Republic of Korea) was added, and the mixture was vortexed for 15 s. The absorbance was measured at 570 nm with a Cary 60 UV/Vis spectrophotometer (Agilent Technologies Inc., Santa Clara, CA, USA). For the determination of *h_tot_*, GB samples at each substrate concentration (4%, 12%, and 20%, *w*/*v*) were subjected to acid hydrolysis with 6 M HCl (Samchun Pure Chemical Co., Ltd., Pyeongtaek, Republic of Korea) in sealed tubes at 95 °C for 31 h with shaking at 50 rpm. After hydrolysis, the mixture was filtered through Whatman No. 1 filter paper, and the filtrate was neutralized with solid calcium carbonate (Showa Chemical Co., Ltd., Tokyo, Japan). The neutralized sample was centrifuged at 4355× *g* for 30 min, and the resulting supernatant was used for *h_tot_* determination. DH was calculated using the following equation [21,22].

(1) $$\mathrm{Degree}\;\mathrm{of}\;\mathrm{hydrolysis}\;\left(\%\right)=\frac{h}{h_{\mathrm{tot}}}\times 100$$

*h*: the extent of hydrolysis estimated from amino-group content.

*h_tot_*: the total amino-group equivalent obtained after acid hydrolysis of the corresponding GB substrate.

Both *h* and *h_tot_* were quantified as L-leucine equivalents using L-leucine (Sigma-Aldrich, St. Louis, MO, USA) as the external standard [22]. Accordingly, the calculated DH represents the extent of hydrolysis estimated by the ninhydrin-based amino-group assay.

### 2.6. Measurement of Viable Cell Counts

Viable cell counts were determined at the end of each fermentation period. A 0.1 mL aliquot of the fermented GB sample was serially diluted in 0.9 mL of 0.85% peptone (Sigma-Aldrich, St. Louis, MO, USA) in water. The diluted samples were spread onto De Man-Rogosa-Sharpe (MRS) agar plates (Difco, Becton Dickinson and Co., Sparks, MD, USA) and incubated at 30 °C for 24 h in an incubator (DI-150, LAB HOUSE, Pocheon, Republic of Korea). The number of colonies formed on the MRS agar plates was counted and expressed as colony-forming units per mL (CFU/mL) of the sample.

### 2.7. Measurement of α-Glucosidase Inhibition

α-glucosidase inhibition (AGI) was measured using a modified version of the method described by Yoon and Kim [16]. The fermented supernatant was used directly for the α-glucosidase assay without prior neutralization. The assay was performed by adding 50 µL of the sample to 50 µL of 0.02 M sodium phosphate buffer (pH 6.8), 200 µL of 2 mM pNPG (4-nitrophenyl α-D-glucopyranoside; Sigma-Aldrich, St. Louis, MO, USA) solution, and 50 µL of α-glucosidase enzyme. The mixture was incubated at 37 °C in a water bath (BFWB 14-6, BNF KOREA, Gimpo, Republic of Korea) for 40 min. After the reaction, 1.5 mL of 0.1 M Na_2_CO_3_ (Duksan Pure Chemicals Co., Ltd., Ansan, Republic of Korea) was added to stop the reaction, and the amount of 4-nitrophenol produced was determined by measuring the absorbance at 405 nm with a Cary 60 UV/Vis spectrophotometer (Agilent Technologies Inc., Santa Clara, CA, USA). The sample blank was prepared under the same assay conditions as the sample reaction, except that heat-inactivated α-glucosidase was used instead of the active enzyme to correct background absorbance from the sample matrix. The α-glucosidase inhibition rate was calculated from the absorbance values, and acarbose (Tokyo Chemical Industry Co., Ltd., Tokyo, Japan) at 1 mg/mL was used as the positive control.

(2) $$\alpha \text{-}\mathrm{glucosidase}\;\mathrm{inhibition}\;\left(\%\right)=\left\{1-\left(\frac{{\mathrm{Abs}}_{\mathrm{Sample}}-{\mathrm{Abs}}_{\mathrm{blank}}}{{\mathrm{Abs}}_{\mathrm{control}}}\right)\right\}\times 100$$

*Abs_sample_* = absorbance of the sample.

*Abs_blank_* = absorbance of the blank.

*Abs_control_* = absorbance of the control.

### 2.8. Statistical Analysis

The Box–Behnken experimental designs, regression analyses, and response surface analyses were performed using XLSTAT-Student version 2024.4.1.1425 software (Addinsoft, Paris, France) in Microsoft Excel version 16.0.18526 (Microsoft Corporation, Redmond, WA, USA). Second-order polynomial models were fitted to the experimental responses, and analysis of variance (ANOVA) was used to evaluate the statistical significance of the fitted models and individual model terms. Model fit was assessed using the coefficient of determination (R^2^) and adjusted R^2^. Multi-response optimization of fermentation was performed by simultaneously maximizing viable cell count and AGI.

The responses obtained experimentally at the model-derived conditions were compared with the corresponding model-predicted values using two-tailed one-sample *t*-tests. Experimental evaluation at each model-derived condition was performed using four replicates (*n* = 4). Statistical significance was defined as *p* < 0.05.

## 3. Results and Discussion

### 3.1. Optimization of Enzymatic Hydrolysis

#### 3.1.1. Model Fitting and Statistical Analysis of Enzymatic Hydrolysis

In the hydrolysis optimization, GB concentration (*X*_1_), enzyme concentration (*X*_2_), and hydrolysis time (*X*_3_) were set as independent variables, and the degree of hydrolysis (DH) was selected as the response (*Y*_1_). A three-factor, three-level Box–Behnken design (BBD, 17 runs) was applied, and the experimental design matrix and observed DH values are presented in Table S1.

The fitted quadratic response surface model for DH was statistically significant (F = 9.32, *p* = 0.004; Table 2). The model explained 92.3% of the observed variation in DH (R^2^ = 0.923), with an adjusted R^2^ of 0.824. However, the lack-of-fit test was significant (*p* = 0.007), indicating that the quadratic model did not fully account for the response variation within the tested range. Accordingly, the model was used primarily to evaluate factor effects and identify conditions associated with high DH within the tested range rather than as a highly precise predictive model.

The final response model equation for the degree of hydrolysis (*Y*_1_, DH) was as follows:

DH (%) = 81.80 + 20.43 *X*_1_ − 0.48*X*_2_ − 1.61*X*_3_ − 6.24*X*_1_*X*_2_ − 2.36*X*_1_*X*_3_ − 12.36*X*_2_*X*_3_ − 9.45*X*_1_^2^
 − 7.88*X*_2_^2^ − 7.63*X*_3_^2^ (3)

Within the fitted empirical model, GB concentration (*X*_1_) exhibited the strongest positive effect on DH (*p* < 0.001). In addition, the quadratic term for GB concentration (*X*_1_^2^) and the interaction between Neutrase concentration and hydrolysis time (*X*_2_*X*_3_) were statistically significant (*p* < 0.05), indicating a nonlinear effect of GB concentration and an interaction between enzyme concentration and hydrolysis time within the tested range. The linear effects of Neutrase concentration (*X*_2_) and hydrolysis time (*X*_3_) were not statistically significant.

The significant lack of fit may reflect response behavior that was not fully represented by the second-order polynomial model. In addition, the model-derived condition associated with the highest predicted DH occurred at the upper boundary of the tested GB concentration range (20%, *w*/*v*), consistent with the strong positive effect of GB concentration within the tested range. Therefore, the present data cannot exclude the possibility of a higher DH response at GB concentrations above 20% (*w*/*v*). However, concentrations above this level were outside the practically workable range under the present processing conditions because homogeneous mixing could not be maintained. Accordingly, the 20% (*w*/*v*) condition represents the model-derived condition associated with the highest predicted DH within the practically feasible range evaluated in this study, and the model was used to describe DH and identify favorable processing conditions only within this range.

#### 3.1.2. Response Surface Analysis

**Figure 1:**
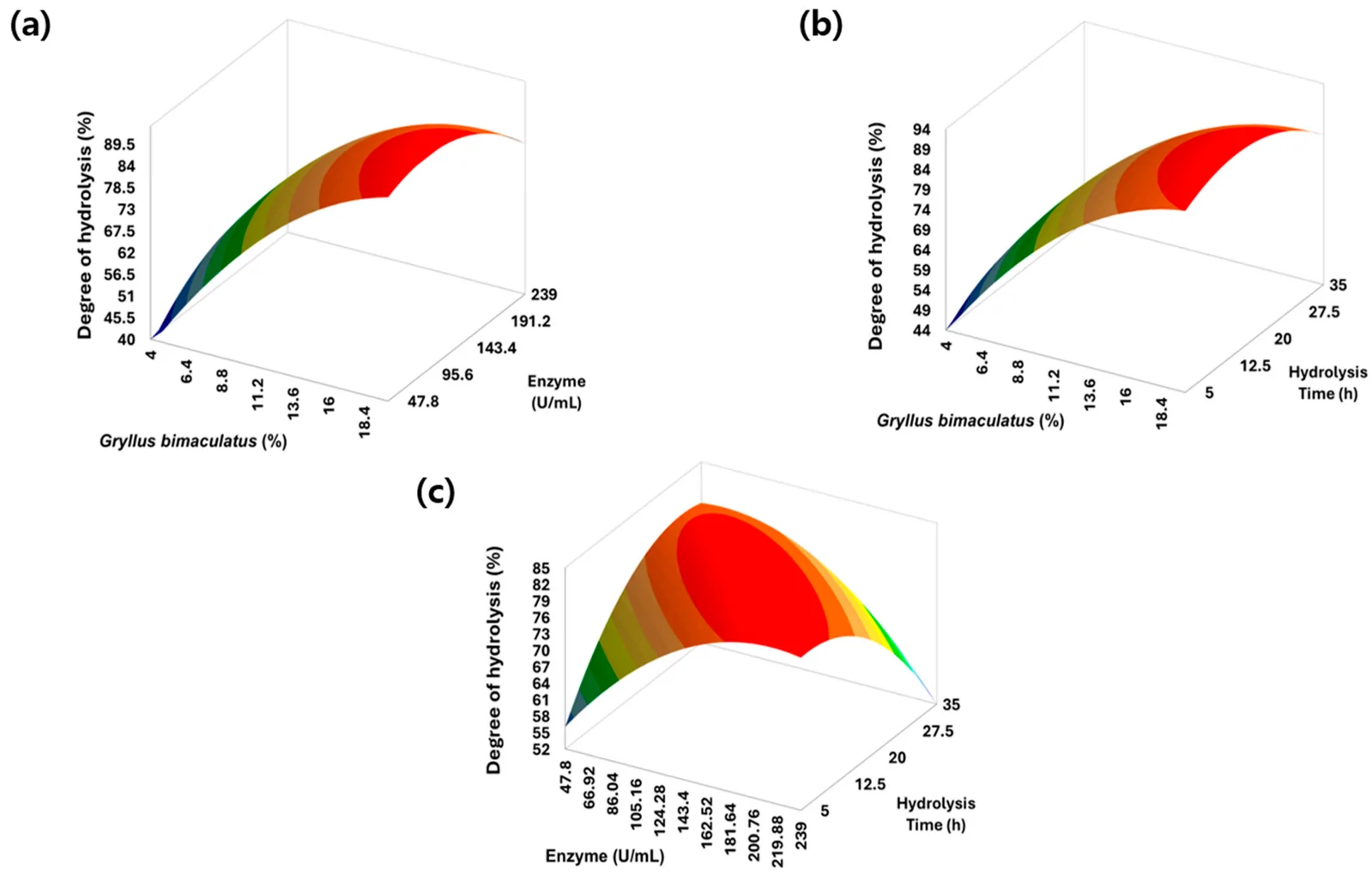
Response surface plots for degree of hydrolysis as a function of (**a**) GB concentration and Neutrase concentration, (**b**) GB concentration and hydrolysis time, and (**c**) Neutrase concentration and hydrolysis time. The color gradient from green to red represents increasing predicted DH values.

To visualize the effects of GB concentration (*X*_1_), Neutrase concentration (*X*_2_), and hydrolysis time (*X*_3_) on DH, three-dimensional response surface plots were generated (Figure 1a–c). These plots illustrate both the individual effects of each variable and their interaction patterns on DH.

As shown in Figure 1a, DH increased markedly with GB concentration, exhibiting a particularly steep slope at low concentrations. At a fixed GB concentration, increasing the Neutrase concentration resulted in only modest changes in DH, suggesting that variation in DH was primarily driven by GB concentration rather than enzyme loading. This trend is consistent with the ANOVA results, in which the linear term of GB concentration (*X*_1_) showed the highest statistical significance.

A similar trend was observed in Figure 1b, where DH increased with GB concentration and hydrolysis time. The response surface exhibited a steeper gradient along the GB concentration axis, whereas changes along the hydrolysis time axis were comparatively moderate. DH increased with hydrolysis time up to a certain point, and then approached a plateau, indicating a diminishing effect of prolonged hydrolysis under the tested conditions. The elongated contour distribution along the GB concentration axis further supports the conclusion that GB concentration exerted a stronger influence on DH than hydrolysis time.

Figure 1c shows the interaction between Neutrase concentration and hydrolysis time. When both variables were at low levels, DH remained relatively low. As Neutrase concentration and hydrolysis time increased simultaneously, DH increased towards an intermediately high response region. Beyond this region, further increases in the two factors were associated with a decrease in predicted DH, indicating a nonlinear response rather than a simple dose-dependent increase. This behavior is consistent with the ANOVA results, in which the interaction term between Neutrase concentration and hydrolysis time (*X*_2_*X*_3_) was statistically significant.

Overall, these response surface profiles indicate that GB concentration is the primary factor influencing DH, while Neutrase concentration and hydrolysis time contribute to DH enhancement within limited ranges before exhibiting diminishing returns. The nonlinear response pattern observed in the quadratic and interaction terms indicates that the effects of the processing variables were not adequately described by simple linear relationships within the tested range.

#### 3.1.3. Experimental Evaluation of the Model-Derived Condition for Enzymatic Hydrolysis

Based on the RSM model, the model-derived condition associated with a high predicted DH within the tested range was 20% (*w*/*v*) GB concentration (*X*_1_), 136.23 U/mL Neutrase concentration (*X*_2_), and 31 h hydrolysis time (*X*_3_), with a predicted DH value of 87.03%. The response obtained experimentally under these model-derived conditions was compared with the predicted value using a two-tailed one-sample *t*-test.

Under the model-derived conditions, the experimental DH was 78.59%, which was lower than the predicted value of 87.03%, although the difference was not statistically significant (*p* = 0.255). However, the significant lack of fit of the quadratic model indicates limitations in its predictive performance across the tested range.

The ninhydrin assay quantifies amino-group-containing compounds rather than peptide-bond cleavage directly [22]. Because the contribution of amino groups present in the untreated GB material was not separately determined, the calculated DH should be interpreted as an assay-based estimate of hydrolysis rather than a direct quantitative measure of peptide-bond cleavage. Furthermore, DH does not provide information on peptide molecular-weight distribution or peptide identity. Therefore, additional peptide profiling would be required to characterize the molecular composition and potential functionality of the resulting hydrolysate.

### 3.2. Optimization of Leuconostoc mesenteroides MKJW Fermentation

#### 3.2.1. Model Fitting and Statistical Analysis of Fermentation

In fermentation optimization, GB concentration (*X*_1_), sucrose concentration (*X*_2_), and fermentation time (*X*_3_) were used as independent variables, while viable cell count (*Y*_1_) and α-glucosidase inhibition (AGI, *Y*_2_) were used as response variables. The experimental design matrix and corresponding response values for the 17 Box–Behnken runs are presented in Table S2.

The fitted second-order polynomial regression models for viable cell count (F = 188.57, *p* < 0.0001) and AGI (F = 14.86, *p* = 0.001) were statistically significant. The viable cell model explained 99.6% of the observed variation (R^2^ = 0.996; adjusted R^2^ = 0.991), whereas the AGI model explained 95.0% of the variation (R^2^ = 0.950; adjusted R^2^ = 0.886). However, significant lack of fit was observed for both models (*p* = 0.0006 for viable cell count and *p* < 0.0001 for AGI; Table 3), indicating that the fitted quadratic equations did not fully account for the response variation within the tested ranges. Accordingly, the models were used primarily to evaluate factor effects and identify conditions associated with high responses within the tested range rather than as highly precise point-prediction models.

The final polynomial equations describing viable cell count (*Y*_1_) and AGI (*Y*_2_) were as follows:

Viable cell count (log CFU/mL) = 9.71 + 0.09*X*_1_ + 0.08*X*_2_ + 2.00*X*_3_ − 0.03*X*_1_*X*_2_ + 0.07*X*_1_*X*_3_
 − 0.02*X*_2_*X*_3_ − 0.02*X*_1_^2^ − 0.19*X*_2_^2^ − 2.43*X*_3_^2^ (4)

AGI (%) = 72.28 − 2.08*X*_1_ + 63.22*X*_2_ + 44.28*X*_3_ + 0.28*X*_1_*X*_2_ + 0.62*X*_1_*X*_3_ + 32.60*X*_2_*X*_3_ −  13.42*X*_1_^2^ − 56.21*X*_2_^2^ − 40.66*X*_3_^2^ (5)

Within the fitted empirical model for viable cell count, fermentation time was the dominant factor: both its linear (*X*_3_) and quadratic (*X*_3_^2^) terms were highly significant (*p* < 0.0001), while the quadratic term for sucrose concentration (*X*_2_^2^) was also significant (*p* = 0.010). In contrast, the linear effects of GB concentration (*X*_1_) and sucrose concentration (*X*_2_) were not significant within the tested range. These results indicate that viable cell growth was primarily influenced by fermentation duration, with an additional nonlinear contribution from sucrose concentration. In the AGI model, the linear and quadratic terms of sucrose concentration (*X*_2_ and *X*_2_^2^) and fermentation time (*X*_3_ and *X*_3_^2^) as well as their interaction (*X*_2_*X*_3_) were statistically significant. This pattern indicates that AGI was strongly associated with both sucrose availability and fermentation duration, with the significant *X*_2_*X*_3_ interaction showing that the effect of one factor depended on the level of the other. In contrast, GB concentration (*X*_1_) showed no significant linear, interaction, or quadratic effect on AGI within the tested range.

The significant lack of fit may reflect nonlinear or higher-order response behavior that was not fully represented by the second-order polynomial models. Unlike a simple physicochemical response, microbial fermentation involves time-dependent changes in cell growth, substrate utilization, and biochemical transformation of the surrounding food matrix [11]. Such dynamic responses may introduce nonlinear or asymmetric behavior that is not fully represented by linear, two-factor interaction, and quadratic terms [12]. In addition, the pure error estimated from the five replicated center point runs was relatively small, particularly for viable cell count (mean square = 0.0010). Therefore, even modest deviations between the fitted surface and observations at non-center design points may result in a statistically significant lack-of-fit test. These findings further support interpreting the models as empirical tools for identifying favorable processing conditions within the tested range rather than as exact predictive equations.

#### 3.2.2. Response Surface Methodology Analysis

**Figure 2:**
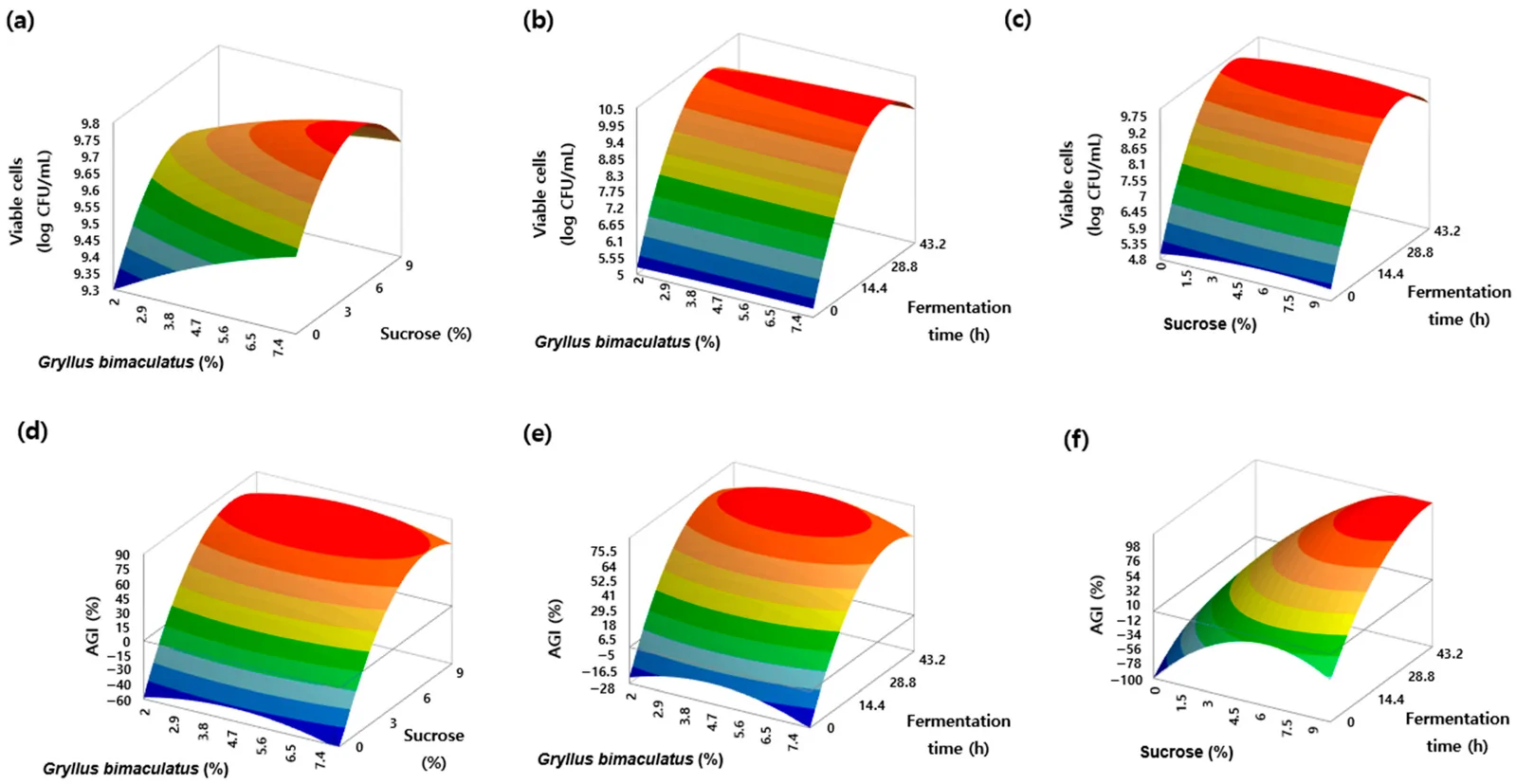
Response surface plots for viable cell counts as a function of (**a**) GB concentration and sucrose concentration, (**b**) GB concentration and fermentation time, and (**c**) sucrose concentration and fermentation time; and α-glucosidase inhibition (AGI) as a function of (**d**) GB concentration and sucrose concentration, (**e**) GB concentration and fermentation time, and (**f**) sucrose concentration and fermentation time. The color gradient from green to red represents increasing predicted response values.

Three-dimensional response surface plots were generated to visualize the effects of GB concentration, sucrose concentration, and fermentation time on viable cell counts (Figure 2a–c).

In Figure 2a, viable cell counts showed relatively limited variation with GB concentration, whereas changes in sucrose concentration produced a curved response rather than a simple linear increase. This pattern is consistent with the ANOVA results, in which the linear effects of both GB concentration (*X*_1_) and sucrose concentration (*X*_2_) were not significant, whereas the quadratic term of sucrose concentration (*X*_2_^2^) was significant. In Figure 2b, the response surface rises steeply along the fermentation time axis and remains relatively flat along the GB concentration axis. This response pattern is consistent with the strong linear and quadratic effects of fermentation time (*X*_3_ and *X*_3_^2^), whereas changes in GB concentration produced no statistically significant linear, quadratic, or interaction effects in the viable cell model. Figure 2c similarly shows a pronounced increase in viable cell count with extended fermentation time, while changes along the sucrose axis were nonlinear but smaller than the time-dependent response. The relatively limited change in surface shape attributable to the combined factors is consistent with the non-significant *X*_2_*X*_3_ interaction for viable cell count.

Taken together, these surfaces indicate that fermentation time was the principal factor associated with viable cell count, whereas sucrose concentration exerted a nonlinear effect and GB concentration had a comparatively limited influence within the tested range.

Response surface plots for AGI are shown in Figure 2d–f. In Figure 2d, the relatively flat GB axis and the steep response along the sucrose axis indicate that AGI was primarily influenced by sucrose concentration, with only a minor contribution from GB concentration. In Figure 2e, AGI increased markedly with fermentation time, with curvature at longer fermentation periods, consistent with the significant linear and quadratic effects of fermentation time. Figure 2f shows a dome-shaped response surface, indicating that AGI depended on the combined levels of sucrose concentration and fermentation time. The curved surface is consistent with the statistically significant interaction between *X*_2_ and *X*_3_ observed in the ANOVA, as well as the significant quadratic effects of both factors.

Based on these response surfaces, a multi-response optimization was conducted to simultaneously maximize viable cell count and AGI. The multi-response analysis identified 7.1% (*w*/*v*) GB, 6.7% (*w*/*v*) sucrose, and 27 h of fermentation as the model-derived condition associated with simultaneously high predicted viable cell count and AGI.

#### 3.2.3. Experimental Evaluation of the Model-Derived Condition

Based on the multi-response RSM analysis, the model-derived condition associated with simultaneously high predicted viable cell count and AGI within the tested range was 7.1% (*w*/*v*) GB concentration (*X*_1_), 6.7% (*w*/*v*) sucrose concentration (*X*_2_), and 27 h fermentation time (*X*_3_). Under these conditions, the predicted values were 9.98 log CFU/mL for viable cell count and 86.57% for AGI.

The responses obtained experimentally under the model-derived conditions were compared with the predicted values using two-tailed one-sample *t*-tests. The experimental viable cell count was 9.94 log CFU/mL, which did not differ significantly from the predicted value of 9.98 log CFU/mL (*p* = 0.249; Table 4), with a difference of only 0.04 log CFU/mL. Thus, the model-derived condition was associated with a high viable cell count within the tested range, although the significant lack of fit indicates that the quadratic model did not fully account for response variation across the tested range.

The experimental AGI was 92.94%, significantly higher than the predicted value of 86.57% (*p* < 0.001; Table 4). Together with the significant lack of fit of the AGI model, this difference indicates limited accuracy for point prediction at the model-derived multi-response condition. Nevertheless, the model remained useful for evaluating the effects of sucrose concentration and fermentation time and for identifying conditions associated with high AGI within the tested range. The lack-of-fit tests assess model adequacy across the tested range, whereas the validation tests assess agreement at a specific model-derived condition. Accordingly, significant lack of fit can coexist with a nonsignificant prediction–observation difference at a validation point, as observed for DH and viable cell count, whereas AGI showed both significant lack of fit and a significant prediction–observation difference.

The different response patterns for viable cell count and AGI indicate that microbial growth and the measured inhibitory response were associated with different processing variables within the investigated range. Fermentation time was the dominant factor affecting viable cell count, with an additional quadratic effect of sucrose concentration, whereas AGI was significantly influenced by sucrose concentration, fermentation time, and their interaction. This distinction was also evident in the experimental results. At 5% (*w*/*v*) GB without added sucrose, viable cell count increased from 5.17 to 9.19 log CFU/mL between 0 and 48 h, whereas AGI remained negative (−69.54% and −83.36%, respectively). In contrast, at 10% (*w*/*v*) sucrose, AGI increased from −31.03% at 0 h to 85.56% at 48 h. These observations indicate that increases in viable cell count were not consistently accompanied by increases in AGI. This distinction is consistent with the time-dependent substrate utilization and biochemical transformation that occur during LAB fermentation [11]. α-Glucosidase-inhibitory activity has been reported in LAB-fermented GB [15], and α-glucosidase-inhibitory peptides have been identified in other edible insect species [8,23]. Fermentation-derived or released peptides may represent one plausible contributor to the observed AGI. However, microbial metabolites, including organic acids, and residual soluble components from the GB matrix or fermentation medium, may also have contributed. Furthermore, previous studies have demonstrated that *L. mesenteroides* MKJW can produce dextran and oligosaccharides during fermentation of other food substrates [16,24]. Given the significant effects of sucrose concentration on AGI in the present study, sucrose-derived carbohydrate products may also have contributed to the observed inhibitory response. However, their production and potential contribution to AGI in the GB fermentation system remain to be determined. Because these components were not individually characterized, their relative contributions could not be distinguished in the present study. Because the fermented supernatant was assayed without prior neutralization, acidification of the fermented system may also have contributed to the measured inhibition. Accordingly, the AGI observed in this study represents an overall in vitro inhibitory response measured in the fermented supernatant, without identifying the compounds responsible for the observed effect.

Further fractionation and compositional analyses would be required to identify the compounds responsible for the observed inhibitory activity. In addition, food-oriented quality and safety attributes were beyond the scope of the present process-optimization study and should be evaluated before practical food application.

## 4. Conclusions

Box–Behnken response surface methodology was used to evaluate and optimize Neutrase-mediated hydrolysis and *Leuconostoc mesenteroides* MKJW fermentation of GB. The model-derived hydrolysis condition associated with a high predicted DH within the tested range was 20% (*w*/*v*) GB, 136.23 U/mL Neutrase, and 31 h, yielding an experimental DH of 78.59%. For fermentation, the model-derived multi-response condition was 7.1% (*w*/*v*) GB, 6.7% (*w*/*v*) sucrose, and 27 h, yielding 9.94 log CFU/mL viable cells and 92.94% in vitro AGI. This study provides process-specific quantitative information on the effects of multiple interacting variables and identifies experimentally evaluated operating conditions for Neutrase-mediated hydrolysis and *L. mesenteroides* MKJW fermentation of GB. These results provide a basis for subsequent compositional, mechanistic, and functional characterization, although the fitted models should be interpreted as empirical optimization tools rather than precise predictive models. Further studies should address peptide and metabolite characterization together with food-relevant safety and quality evaluation to determine the applicability of the processed GB materials as food ingredients.

## Figures and Tables

**Table 1:** Independent variables and their coded levels used in the Box–Behnken designs for enzymatic hydrolysis and fermentation of *Gryllus bimaculatus*.

Process	Independent Variable	Symbol	Levels		
			−1	0	1
Hydrolysis	GB concentration (% *w*/*v*)	*X*_1_	4	12	20
	Enzyme concentration (U/mL)	*X*_2_	47.80	143.40	239.00
	Hydrolysis time (h)	*X*_3_	5	20	35
Fermentation	GB concentration (% *w*/*v*)	*X*_1_	2	5	8
	Sucrose (% *w*/*v*)	*X*_2_	0	5	10
	Fermentation time (h)	*X*_3_	0	24	48

**Table 2:** Analysis of variance (ANOVA) for the fitted quadratic model of degree of hydrolysis (DH) in Neutrase hydrolysis of *Gryllus bimaculatus*.

Source	Sum of Squares	df	Mean Square	F-Value	*p*-Value
Model	5135.56	9	570.62	9.315	0.004
*X*_1_	3339.92	1	3339.92	54.52	0.0002
*X*_2_	1.83	1	1.83	0.03	0.87
*X*_3_	20.70	1	20.70	0.34	0.58
*X*_1_ *X*_2_	155.80	1	155.80	2.54	0.15
*X*_1_ *X*_3_	22.18	1	22.18	0.36	0.57
*X*_2_ *X*_3_	610.96	1	610.96	9.97	0.02
*X*_1_^2^	449.81	1	449.81	7.34	0.03
*X*_2_^2^	289.42	1	289.42	4.72	0.07
*X*_3_^2^	244.95	1	244.95	4.00	0.09
Lack of fit	402.48	3	134.16	20.48	0.007
Pure error	26.21	4	6.55	–	–

*X*_1_ = GB concentration; *X*_2_ = Neutrase concentration; *X*_3_ = Hydrolysis time.

**Table 3:** Analysis of variance (ANOVA) for the fitted quadratic models of viable cell counts and α-glucosidase inhibition (AGI) in *Leuconostoc mesenteroides* MKJW fermentation of *Gryllus bimaculatus*.

Source	df	Viable Cells		AGI	
		F-Value	*p*-Value	F-Value	*p*-Value
Model	9	188.57	<0.0001	14.86	0.001
*X*_1_	1	1.969	0.203	0.06	0.81
*X*_2_	1	1.579	0.249	57.17	0.0001
*X*_3_	1	942.852	<0.0001	28.05	0.001
*X*_1_ *X*_2_	1	0.076	0.791	0.001	0.98
*X*_1_ *X*_3_	1	0.499	0.503	0.003	0.96
*X*_2_ *X*_3_	1	0.055	0.822	7.60	0.03
*X*_1_^2^	1	3.517	0.103	1.36	0.28
*X*_2_^2^	1	12.503	0.010	23.79	0.002
*X*_3_^2^	1	734.111	<0.0001	12.45	0.01
Lack of fit	3	76.17	0.0006	488.73	<0.0001
Pure error ^a^	4	–	–	–	–

*X*_1_ = GB concentration; *X*_2_ = sucrose concentration; *X*_3_ = fermentation time. ^a^ Pure error (df = 4) is the replicate variance among the five center-point runs and served as the denominator of the lack-of-fit F-test; it therefore has no associated F- or *p*-value. Sum of squares/mean square were 0.0041/0.0010 for viable cells and 10.63/2.66 for AGI, giving lack-of-fit F-values (mean square of lack of fit/mean square of pure error) of 76.17 and 488.73, respectively, consistent with the values reported above.

**Table 4:** Model-predicted and experimentally observed values of the response variables at their respective model-derived conditions.

Response Variable	Optimal Condition	Predicted	Experimental	*p*-Value ^a^
Degree of hydrolysis, DH (%)	20% (*w*/*v*) GB, 136.2 U/mL Neutrase, 31 h	87.03	78.59 ± 11.40	0.255
Viable cell count (log CFU/mL)	7.1% (*w*/*v*) GB, 6.7% sucrose (*w*/*v*), 27 h	9.98	9.94 ± 0.05	0.249
α-Glucosidase inhibition, AGI (%)	7.1% (*w*/*v*) GB, 6.7% sucrose (*w*/*v*), 27 h	86.57	92.94 ± 0.50	<0.001

^a^ *p*-values were obtained using two-tailed one-sample *t*-tests comparing the experimentally observed values with the corresponding model-predicted values (*n* = 4 replicates per response).

## Data Availability

The data supporting the findings of this study are available from the corresponding author upon reasonable request. The data are not publicly available due to intellectual property considerations.

## Supplementary Materials

The following supporting information can be downloaded at https://www.mdpi.com/article/10.3390/foods15203684/s1. Table S1: Box-Behnken design (BBD) matrix of independent variables and results of Neutrase hydrolysis of *Gryllus bimaculatus*; Table S2: Box-Behnken design (BBD) matrix of independent variables and results of *Leuconostoc mesenteroides* MKJW fermentation of *Gryllus bimaculatus*.
