# Supplementary material for: Optimization of Enzymatic Hydrolysis and Leuconostoc mesenteroides MKJW Fermentation of Gryllus bimaculatus Using Response Surface Methodology
Source: Foods. 2026 Oct 16;15(20):3684. doi: 10.3390/foods15203684 (PMC13704338; doi:10.3390/foods15203684)
Supplement: Supplementary file 1 [file foods-15-03684-s001.zip › foods-4577088-supplementary.pdf]

Supplementary Materials

Optimization of Enzymatic Hydrolysis and *Leuconostoc mesenteroides* MKJW Fermentation of *Gryllus bimaculatus* Using Response Surface Methodology

Minji Cho, Misook Kim

Table S1. Box-Behnken design (BBD) matrix of independent variables and results of Neutrase hydrolysis of *Gryllus bimaculatus*.

Run	GB concentration (%, X_1)	Neutrase concentration (U/mL, X_2)	Hydrolysis time (h, X_3)	Degree of hydrolysis (%, Y_1)
1	4	47.80	20.00	31.35
2	20	47.80	20.00	84.97
3	4	239.00	20.00	56.46
4	20	239.00	20.00	85.12
5	4	143.40	5.00	41.65
6	20	143.40	5.00	86.95
7	4	143.40	35.00	47.21
8	20	143.40	35.00	83.09
9	12	47.80	5.00	64.85
10	12	239.00	5.00	75.02
11	12	47.80	35.00	82.28
12	12	239.00	35.00	43.02
13	12	143.40	20.00	83.85
14	12	143.40	20.00	80.70
15	12	143.40	20.00	84.64
16	12	143.40	20.00	81.56
17	12	143.40	20.00	78.24

Table S2. Box-Behnken design (BBD) matrix of independent variables and results of *Leuconostoc mesenteroides* MKJW fermentation of *Gryllus bimaculatus*.

Run	GB concentration (%, X_1)	Sucrose concentration (%, X_2)	Fermentation time (h, X_3)	Viable cells (log CFU/mL, Y_1)	AGI (%, Y_2)
1	2.00	0.00	24.00	9.12	-73.22
2	8.00	0.00	24.00	9.37	-70.65
3	2.00	10.00	24.00	9.68	75.38
4	8.00	10.00	24.00	9.82	79.07
5	2.00	5.00	0.00	5.23	-38.32
6	8.00	5.00	0.00	5.27	-51.02
7	2.00	5.00	48.00	9.11	86.18
8	8.00	5.00	48.00	9.41	75.96
9	5.00	0.00	0.00	5.17	-69.54
10	5.00	10.00	0.00	5.03	-31.03
11	5.00	0.00	48.00	9.19	-83.36
12	5.00	10.00	48.00	8.97	85.56
13	5.00	5.00	24.00	9.69	69.48
14	5.00	5.00	24.00	9.66	73.21
15	5.00	5.00	24.00	9.71	73.60
16	5.00	5.00	24.00	9.73	72.71
17	5.00	5.00	24.00	9.74	72.41
